# Integrating nursing activities into general practices in Switzerland: a mixed-methods study

**DOI:** 10.1017/S146342362510039X

**Published:** 2025-09-29

**Authors:** Muriel Schütz Leuthold, Joëlle Schwarz, Fatima El Hakmaoui, Renzo Scuderi, Nicolas Senn, Christine Cohidon

**Affiliations:** Department of Family Medicine, Center for Primary Care and Public Health, University of Lausanne, Lausanne, Switzerland

**Keywords:** case management, general practice, mixed-methods, nurse, primary care

## Abstract

**Aim:**

The aim of this study is to describe the rollout of nursing activities during the pilot project’s first 12 months (2019–2021), especially relating to what was initially planned in the nurses’ job description.

**Background:**

To provide more comprehensive services and reinforce primary care, a pilot implementation study assessed the integration of nursing activities into eight general practitioners’ (GPs’) practices. The study evaluated how new types of activities were integrated and rolled out over the first year.

**Methods:**

A mixed-methods observational study collected quantitative data on nursing activities and duration and qualitative data via five interviews with nurses and patients and one focus group with six GPs. Investigators combined quantitative and qualitative data in discussions about their results.

**Results:**

New nursing activities were rolled out progressively, especially follow-up activities with chronically ill patients, with a median time dedicated/month of 21h58 (range: 9h25 to 64h50) at six months and 48h43 (range: 11h01 to 59h51) at 12 months. One-off clinical activities are more easily integrated: the median time dedicated/month was 40h01 (range: 13h44 to 74h53) at six months and 40h30 (range: 9h38 to 76h51) at 12 months. Three elements were crucial in the implementation of nursing activities. The nurse’s previous professional experience influenced the scope of activities developed. GPs’ willingness to refer patients to the nurses enabled the latter to carry out follow-up activities with care plan. Lastly, the implementation of nursing activities was also made possible by patients’ acceptance of being cared for by nurse instead of a GP.

**Conclusion:**

Implementation of nursing activities increased progressively, although more slowly for activities with chronically ill patients and within care plans, principally due to the overall change faced by GPs and nurses.

## Introduction

Faced with growing healthcare needs and expenditures due to ageing populations and increasing numbers of patients with chronic conditions, many high-income countries are reorganising their healthcare systems to reduce the fragmentation in overall care delivery and reinforce primary care provision (Kringos *et al.* 2015; Polin *et al.* 2021). One of the main strategies used to ensure the continuity of care and reduce that fragmentation of care for patients with complex healthcare needs is improving the coordination of care (Penm *et al.* 2017; Stille *et al.* 2005). Improving care coordination is a complex process requiring major changes in how the provision of care is organised. Organisational interventions described in the literature concern several areas, such as setting up multidisciplinary PC teams (Powell Davies *et al.* 2006). The latter usually integrate other healthcare professionals, such as nurses, pharmacists, or social assistants (Cohidon and Senn 2023; Saint-Pierre, Herskovic, and Sepulveda 2018; Ramond-Roquin, Allory, and Fiquet 2020) and new organisational elements that assist integration, such as case management or care planning (Edwards, Dorr, and Landon 2017; Cardinaux *et al.* 2020).

Nurses’ roles and activities in these new organisations vary between countries depending on their healthcare system’s general organisation and policy commitments. They help to respond to this sector’s needs via two mechanisms: the diversification of care delivery and the transfer of tasks from GPs to nurses, especially when involving advanced practice nurses. Diversified care delivery has come through new nursing activities developed in the fields of health promotion and the follow-up of patients with chronic conditions (Pelletier *et al.* 2019; Stephen, McInnes, and Halcomb 2018). In contexts where tasks are transferred away from GPs, nurses can perform certain diagnostic acts and even prescribe treatments within well-defined frameworks (Bourgueil, Marek, and Mousques 2008).

Working rarely in interprofessional organisation, GPs’ practices in Switzerland require also some organisational changes to face the challenges of our healthcare system (Senn, Ebert, and Cohidon 2016). To this end, various pilot projects aimed at including other professionals, especially nurses in GPs’ practices have recently emerged in Switzerland. They show promising results. However, implementing them on a large scale and in the long term remains challenging due to legal frameworks and financing mechanisms. (Altermatt-von Arb *et al.* 2023; Gysin *et al.* 2019; Walger *et al.* 2024)

To strengthen primary care and enhance care coordination in GPs’ practices, a two-year pilot project was carried out in eight practices across the canton of Vaud, in Switzerland (Schutz Leuthold *et al.* 2020). This project consisted in integrating registered nurses into GPs’ practices, developing new activities, including case management with an individual care plan. This project is the result of a collaboration of the canton of Vaud’s public health authorities, who fully funded the nurses, and the Department of Family Medicine (DFM) at Unisanté.

## Aim of the study

This work aims to describe the implementation of nursing activities during the pilot project’s first 12 months (2019–2021), especially relating to what was initially planned in the nurses’ job description. Thus, our rationale was to assess the progress and influencing factors of transitions and change at the critical point of one year.

## Methods

### Study design

To describe the rollout of these nursing activities, we conducted a mixed-methods approach, collecting and analysing both quantitative and qualitative data through a convergent design. Mixed methods were particularly appropriate as they provided a deep understanding of the intervention’s complexity in primary care research (Guetterman, Fetters, and Creswell 2015; Vedel *et al.* 2019). The methods, results, and discussion set out below were prepared according to the STROBE guidelines for reporting observational studies (von Elm *et al.* 2014) and the SRQR guidelines for reporting qualitative research (O’Brien *et al.* 2014; Tong, Sainsbury, and Craig 2007).

### Study setting

The study occurred in eight GPs’ practices (Table 1) employing a total of 20 full-time equivalent (FTE) GPs in the canton of Vaud. GPs’ practices entered the scheme progressively between July 2019 and March 2020, and the study focused on the one-year period from their entry date. This period was marked by the first wave of COVID-19, during which non-emergency care activities partially stopped. The selection criteria for practices were their interest in the project, their location (to ensure diversity across urban and rural areas), and their workforces (to ensure solo and group practices).

**Table 1.** Practices’ characteristics before entry into the pilot project

Practices	Workforce (FTEs)	Location
1	0.6 nurses, 3.1 GPs, 5.4 MAs*	Semi-rural
2	0.6 nurses, 3.3 GPs, 4.1 Mas	Rural
3	0.4 nurses, 3.3 GPs, 3 MAs	Semi-rural
4	0.8 GP, 1 MAs	Semi-rural
5	3.9 GPs, 3.9 MAs	Rural
6	2.1 GPs, 1.1 MAs	Urban
7	3.3 GPs, 3.1 MAs	Semi-rural
8	2.7 GPs, 4.4 MAs	Urban

MAs*: medical assistants.

### Quantitative study

#### Population

Anonymised quantitative data on nurses’ activities were collected for all patients in the eight GPs’ practices.

#### Data

Nurses’ activities were collected through a web application developed specifically for the project. This consisted of a pre-established list of practice activities in four categories, prepared with the GPs and nurses involved in the pilot project (Table 2).

**Table 2.** Nursing activities per categories

Category	Activities
**Coordination activities within a care plan**	Coordination within the network
	Developing care plans
	Updating care plans
	Practice staff meetings
**Clinical activities within a care plan**	Contact with patients
	First nursing visits
	Medical team meetings with patients
	Follow-up nursing visits
**Clinical activities without a care plan/one-off**	Contact with patients
	Clinical consultations for acute care
	Clinical consultations for follow-up care
	Preventive consultations
	Coordination within the network
	Practice staff meetings
	Emergencies
	Home visits
	COVID-19 activities
**Non-patient activities**	Invoicing
	Developing quality of care procedures

Nurses recorded in the web application their daily activities and specified the types and categories of those activities and how long they took throughout the pilot project’s first year. SLM and EHF monitored data completion throughout the pilot project and supported nurses in the data collection.

#### Analyses

To analyse the distribution and evolution of nursing activities over time, we calculated average time and range per month for each category of nursing activities. Additionally, the numbers of patients managed by nurses each month over this period was assessed to link them with the time dedicated to the activities. We used StataSE V.16. software to calculate standard descriptive statistics.

### Qualitative study

#### Population

For convenient reason, qualitative data were collected in five GPs’ practices illustrating the specificities and characteristics of all eight GPs’ practices in term of human resources, location, and activities. In each practice, the data collection involved GPs, nurses, and patients (Table 3) and was performed by two researchers not involved in implementing the pilot project (SLM and SJ). SLM recruited volunteer nurses and GPs by email to take part in interviews. Nurses recruited face-to-face volunteer patients who gave a written informed consent to participate to interviews.

**Table 3.** Characteristics of qualitative study participants

Participants	Gender F/M	Age (range)
Nurses	4/1	31 – 45
GPs	5/1	36 – 48
Patients	3/2	52 – 70

#### Data

Data collection occurred from December 2020 to April 2021. JS conducted 90-minute face-to-face in-depth semi-structured interviews with five nurses. SLM conducted 30–50-minute face-to-face in-depth semi-structured interviews with five patients benefiting from nursing follow-up. Interviews were conducted in a GP’s consultation room. SLM and SJ jointly conducted the focus group discussion (FGD) with six GPs via a videoconference lasting approximately 90 minutes. Interviews with nurses and the FGD with GPs collected information regarding practice organisation, functioning, the delegation of tasks, and staff satisfaction. Information collected through patient interviews included the satisfaction of nursing follow-up and the impact on their self-empowerment and health, their experience regarding care coordination, and their global satisfaction. Interview guides were specially developed for the study by SLM, reviewed and internal tested by SJ, CC, and EHF (Kallio *et al.* 2016). All interviews were audio recorded.

#### Analyses

Audio recordings of the FGD and interviews were transcribed in full. To ensure confidentiality, identifying data were removed. Transcripts were imported in MAXQDA software (V.20). We performed a thematic analysis, using an inductive approach, to extract categories, assemble these into themes (Thomas 2006; Gale *et al.* 2013). SLM analyses all qualitative data. EHF coded 20 percent of the data. Emerging categories and themes coded by both researchers were discussed and adapted within the research team (SLM, EHL, SJ, and CC), including an expert in qualitative research (SJ). SLM established a coding book and adapted it through the analysis process.

## Results

The findings of our quantitative and qualitative analyses are presented jointly under the two main themes of the rollout of nursing activities and the factors influencing that rollout.

### Nursing activities

During the first year of the pilot project’s implementation, nine nurses (5.75 FTE positions) recorded 12,862 activities performed with 2,359 patients managed. The median activity duration was 30 minutes (range: 2 to 510).

**Figure 1.**
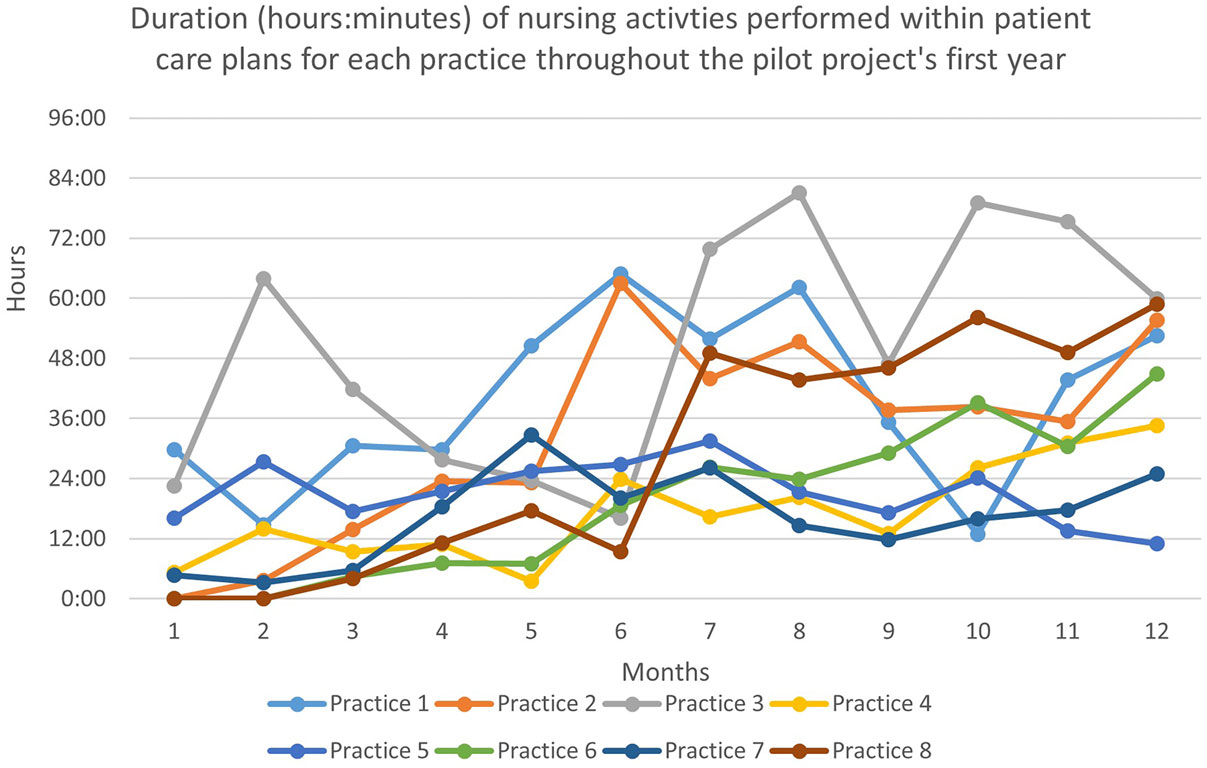
Duration (hours:minutes) of nursing activities performed within patient care plans during the pilot project’s first year *(the first wave of COVID-19 struck in April 2020, which was project implementation month 10 for practices 1 and 2, month 5 for practices 3 and 4, month 4 for practice 5, month 3 for practice 7, and month 2 for practices 7 and 8).*

As Figure 1 illustrates, we observed a general 12-month increase, across all the practices but one (practice 5), in the monthly duration of the activities performed by nurses within a care plan. At six months, the median monthly time dedicated to activities within a care plan was 21 h 58 (range: 9 h 25 to 64 h 50 across nurses). At 12 months, the median time was 48 h 43 (range: 11 h 01 to 59 h 51). The rollout of activities occurred more comprehensively in some GPs’ practices (e.g., practices 1, 2, 3, and 8) than in others (e.g., practices 5 and 7).

Figure 1 also shows a decrease in activities during the first wave of COVID-19. Between March and May 2020, nurses performed activities linked to COVID-19 (El Hakmaoui and Cohidon 2021).

**Figure 2.**
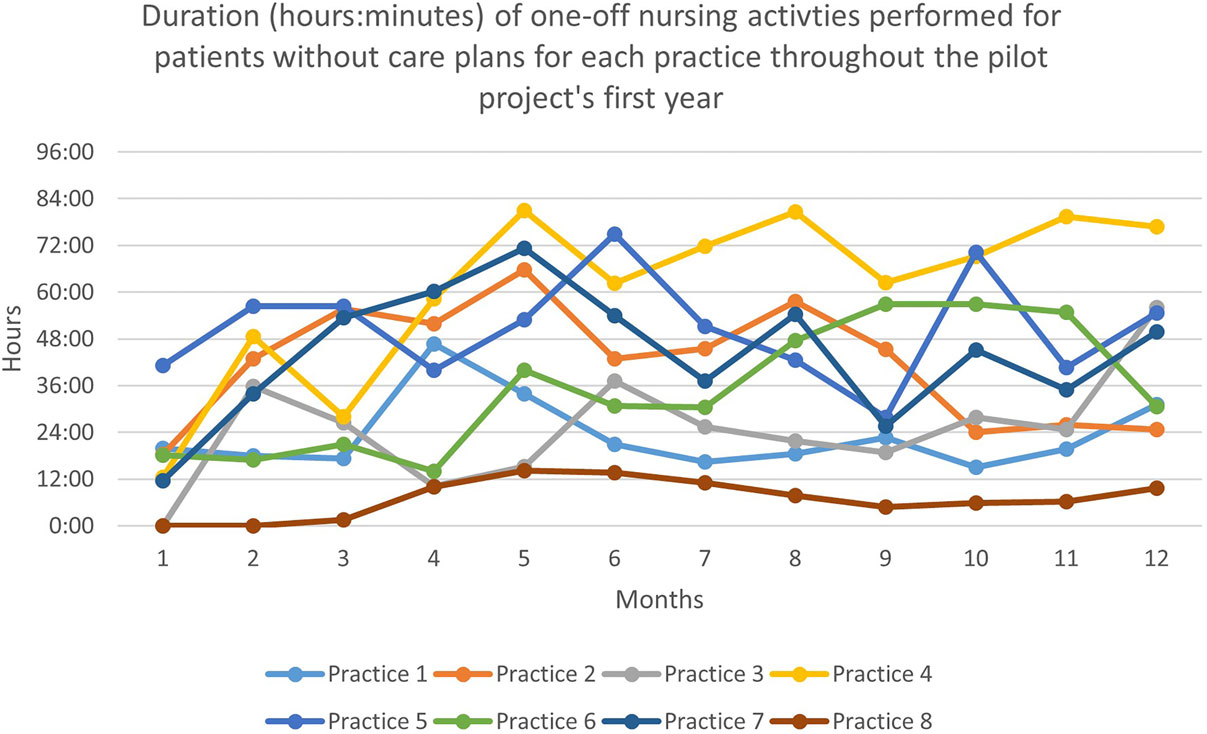
Duration (hours:minutes) of one-off nursing activities perfomed for patients without care plans during the pilot project’s first year *(the first wave of COVID struck in April 2020, which was project implementation month 10 for practices 1 and 2, month 5 for practices 3 and 4, month 4 for practice 5, month 3 for practice 7, and month 2 for practices 7 and 8).*

As Figure 2 shows, the monthly duration of one-off activities performed for patients without care plans increased faster but stopped increasing after six months. The median time dedicated to these activities was 40 h01 (range: 13 h 44 to 74 h 53 across nurses) at six months and 40 h 30 (range: 9 h 38 to 76 h 51) at 12 months. The general evolution of one-off activities was more irregular and quite up and down.

### Qualitative interviews analysis

Qualitative data from nurses’ interviews confirmed the monitoring data and highlighted a general increase in the implementation of nursing activities within patients’ care plans, particularly follow-up activities such as secondary prevention and therapeutic education. ‘*In fact, you move gradually, because first, you need to figure out how you can best support the patients’.* Most nurses also described how, even after a year, the distribution of activities was still evolving: ‘*[My job description] will continue to evolve’.*

Several issues explaining this rollout emerged from interviews including nursing background, GPs’ willingness to refer patients and patient acceptance.

#### Nurses’ professional experience, skills, and knowledge

Nurses perceived their diverse prior professional experiences to be an advantage regarding their work in GPs’ practices: ‘*For me, having diverse professional experience is a great asset, […] which leads me to see lots of possibilities, in fact’.* Nurses’ prior professional experience also influenced the scope of the activities they implemented. For example, nurses with professional experience in emergency care dealt with some of their practice’s emergencies: *‘I took care of some of the emergencies—right from the start—because I have a lot of experience in emergencies’.*

On the contrary, a lack of skills and experience in certain domains were considered barriers, such as secondary prevention and therapeutic education: *‘I don’t consider myself as qualified as an addictologist for giving this kind of* [tobacco cessation] *support’.* ‘*In fact, you move gradually, because first, you need to figure out how you can best support the patients’.*

#### GPs’ willingness to refer patients to nurses

We also found that GPs’ willingness to refer patients to nurses for follow-up within a care plan was a major influencing factor in the rollout of activities. Indeed, all the patients interviewed were referred to a nurse by a GP. But several factors influenced GPs’ willingness to refer patients for nursing follow-up.

Firstly, the lack of existing interprofessional collaboration within GPs’ practices and the fact that GPs had previously been taking care of patients on their own were barriers to their willingness to delegate tasks to nurses. As one doctor said, *‘I think* [the difficulty delegating] *is also a question of practice, of medical culture’.*

Interviewed nurses described GPs’ trust in their skills as another factor influencing task delegation. Nurses mentioned having to practically demonstrate their skills and gain GPs’ trust. This was confirmed by one GP who expressed how, *‘as time passes, we see [her] range of nursing skills more and more clearly, and then the range of collaboration that takes place’.*

Nurses and GPs both identified two other factors: GPs’ knowledge of their patients and GPs’ work schedules. It was easier for GPs to identify and refer patients for follow-up within a care plan when they had a comprehensive overview of their case. Furthermore, they were keener to refer patients to nurses when they had a busy schedule, as one nurse explained: *‘If it is a more experienced doctor, you tend to cooperate more with one another because he has greater knowledge about his patients and a busier schedule’.* GPs affirmed this*: ‘My two colleagues, who use* [nurses’ support] *the least, are those who have recently started medical practice, and they are not fully booked yet’.*

### Patient acceptance

Adding another healthcare professional, i.e. a nurse, to the organisation of care was generally well perceived by patients. However, nurses assessed that the potential initial reticence to being managed by a nurse was related to the novelty of this idea: *‘We had a few* [patients] *who found it difficult to accept* [nursing follow-up]. *However, it’s often enough that they meet me* [a first time in order to initiate a therapeutic relationship]’.

Nevertheless, patients considered the quality of care provided by nurses to be equivalent to that provided by GPs, as illustrated by the following two patient quotes: *‘For me, the care provided by a nurse is as good as that of a doctor’.*

Moreover, the patients appreciated the relationship developed with their nurses and those nurses’ human qualities, which they found validating, supportive and motivating. ‘*With the nurse, it’s simpler, more human, like a family member who explains everything to you’.* Some patients also reported feeling more comfortable talking with nurses about certain health issues: *‘With a nurse, I think* […] *that the patient will say more than with the doctor.* […] *for me, yes’.* They also appreciated nurses’ availability and accessibility: *‘I think that it’s good with the nurse because she takes the time to explain things clearly’.*

## Discussion

The present findings showed that the rollout of nursing activities, especially activities performed within the framework of a care plan, generally progressed throughout the pilot project’s first year. This process was influenced by several factors, such as nurses’ skills and experience, GPs’ willingness to refer patients and patient acceptance.

The nursing role is still relatively unknown in Switzerland (Gysin *et al.* 2019) and registered nurses often have similar roles to medical assistants (Josi and Bianchi 2019). Nurses in the MOCCA project therefore did not have a model to rely on to develop their role and activities, which explains the slower uptake for more specific activities such as case management. This gradual increase can also be attributed to the time nurses took to acquire skills and experience in family medicine care. This acquisition was faster when nurses had previous experience in a primary care (PC) setting. Training is also crucial for nurses to gain confidence in their role and activities (Busca *et al.* 2021; Drennan *et al.* 2011; McCullough *et al.* 2020). In the absence of pre-graduate training, it is necessary to implement early training to support nurses.

Several studies also corroborate that teams working (e.g. nurses) in GPs’ practices depend on the task delegation by GPs (Condon, Willis, and Litt 2000; Willis, Judith, and Litt 2000; Finlayson and Raymont 2012; Jaruseviciene *et al.* 2013). Finlayson *et al*. describe how 68% of nursing work in GPs’ practices was delegated by a GP (Finlayson and Raymont 2012). Other studies have also identified that nurses rely on the workflow of GPs (McInnes *et al.* 2015; Condon, Willis, and Litt 2000). Due to the fee-for-time payment system of the Swiss PC system, nurses have also encountered this issue. This highlights the limitations of this payment system in transforming private healthcare structures into multiprofessional organisations, especially in the initial stages. Alternative payment systems, such as bundled payment system or blended payment system are more appropriate for such organisation (Nolte and Woldmann 2021; Feldhaus and Mathauer 2018; Edwards *et al.* 2014).

Another key element facilitating the integration of nursing activities, which was also identified in our study, is trust among professionals. Several authors have also identified trust as a key element of collaboration (Pullon 2008); it builds over time and depends (among other things) on the development of mutual understanding (Bradley, Ashcroft, and Noyce 2012). Other studies have also shown that even if GPs trust nurses and recognise their skills and added value, some have difficulty sharing care responsibilities with them (Willis, Judith, and Litt 2000; Pullon 2008). Interprofessional education (IPE) can help address this issue (Karam *et al.* 2021). In the context of the MOCCA project, IPE was limited to a single day and did not involve all teams. Strengthening IPE at the beginning of the project is clearly necessary.

Finally, at patients’ level, the apprehension of the unknown is quickly overshadowed by the satisfaction of the quality of nursing care. Patients are generally just as satisfied with the care provided by a nurse as they were with that provided by a GP (Laurant *et al.* 2018). However, it is necessary to work on the understanding of the nursing role with patients. Doctors are best positioned to do it.

### Strengths and limitations

The present study had several strengths, including its real-world conditions and the mixed-methods approach used to contextualise the implementation of activities. Furthermore, qualitative data from multiple perspectives (nurses, GPs, and patients) allowed for a comprehensive exploration of experiences and perceptions within GPs’ practices. However, limitations were also present. Nurses self-reported their activities, potentially introducing memory bias, and patients interviewed may have been subject to selection bias as only patients who agreed to nurse follow-up were included. Social desirability bias may also have influenced qualitative data. Further evaluation of this two-year pilot project is needed to confirm these initial findings and provide a deeper understanding of the implementation of nursing activities and influencing factors.

## Conclusion

The rollout of new nursing activities into GPs’ practices was a progressive increase in the number of nursing activities, principally for the delivery of nurses’ patient follow-up activities. These results highlight the importance of early nursing training to assume their new role, as well as training staffs in interprofessionality.

## Data Availability

The data are stored at the institution. Datasets generated and analyzed during the current study are not publicly available. At this point, researchers are still working on the material. However, it is available from the corresponding author on a reasonable request.
